# Burnout, working conditions and gender - results from the northern Sweden MONICA Study

**DOI:** 10.1186/1471-2458-10-326

**Published:** 2010-06-09

**Authors:** Sofia Norlund, Christina Reuterwall, Jonas Höög, Bernt Lindahl, Urban Janlert, Lisbeth Slunga Birgander

**Affiliations:** 1 Department of Public Health and Clinical Medicine, Occupational Medicine, Umeå University, Umeå, Sweden; 2 Department of Sociology, Umeå University, Umeå, Sweden; 3 Department of Public Health and Clinical Medicine, Epidemiology, Umeå University, Umeå, Sweden; 4 Research and Development Department, Jämtland County Council, Östersund, Sweden

## Abstract

**Background:**

Sick-leave because of mental and behavioural disorders has increased considerably in Sweden since the late nineties, and especially in women. The aim of this study was to assess the level of burnout in the general working population in northern Sweden and analyse it's relation to working conditions and gender.

**Methods:**

In this cross-sectional study the survey from the MONICA-study (Monitoring of Trends and Determinants in Cardiovascular Disease) in northern Sweden 2004 was used. A burnout instrument, the Shirom Melamed Burnout Questionnaire (SMBQ), was incorporated in the original survey which was sent to a random sample of 2500 individuals with a response rate of 76%. After including only actively working people, aged 25-64 years, our study population consisted of 1000 participants (497 women and 503 men). ANOVA and multiple linear regression models were used.

**Results:**

The prevalence of a high level of burnout (SMBQ >4.0) was 13%. Women had a higher level of burnout than men with the most pronounced difference in the age group 35-44 years. In both sexes the level of burnout decreased with age. Demand and control at work, and job insecurity were related to burnout. In women the level of education, socioeconomic position, work object, and working varying hours were of importance. Interaction effects were found between sex and work object, and sex and working hours. In a multiple regression analysis almost half of the gender difference could be explained by work related and life situational factors.

**Conclusions:**

Working life conditions contributed to the level of burnout in this actively working sample from the general population in northern Sweden. Especially in women, socioeconomic position was associated with burnout. The high level of burnout in women compared to men was partly explained by more unfavourable working conditions and life situational factors. Efforts to level out gender differences in burnout should probably focus on improving both working and socioeconomic conditions for women.

## Background

Burnout can be described as a condition resulting from a continuous draining of a person's energy resources [1]. Chronic strain without recurrent physical and mental recovery can lead to illness, disease and burnout. Common symptoms in burnout are physical and psychological fatigue, emotional exhaustion, cognitive weariness, sleep disturbances, depression, and anxiety [2,3]. Insufficient sleep has been presented as an important factor and can both precede and coincide with burnout [4]. Severe burnout is associated with a considerably increased risk for all-cause sickness absence [5]. In Sweden long-term sick-leaves due to mental and behavioural disorders have increased considerably from the late nineties, especially in women [6].

Maslach originally described burnout as a condition related to work and especially prevalent in occupations with client-related tasks. The burnout concept has since been expanded to include all professions [7]. Studies show that organizational and work related situations contribute to stressful, untenable positions and probably also to burnout [8-10]. However, longitudinal studies have mainly focused on mental ill health and not on the specific burnout syndrome [11]. There is a considerable over-lap between burnout and mental illness [12]. In a prospective study on dentists a reciprocal relationship between burnout and depression was found, and the relationship between job strain and depression was mediated by a burnout state [13]. A work situation distinguished by an effort-reward imbalance and personal overcommitment also coincided with burnout [14,15]. Personality traits may be important in the burnout process [16,17]. Coping resources and abilities determine how the individual reacts in certain situations and deals with problems.

Most articles published on the burnout phenomenon have been based on specific professions. There are few population-based studies that deal with the impact of work, gender and other life factors on burnout [8,18-20]. Life in general contributes to the stress burden. Work and private life have also become more integrated and influence each other [10]. Even persons outside the labour market may experience high levels of burnout symptoms. In some studies marital status has been shown to be a significant factor related to burnout while other studies have found no such association. Non married men and divorced women have been proposed to be possible risk groups [18,19].

Information on burnout occurrence in the general working population is fundamental for understanding the context in which the problem may appear and how to develop preventive strategies. Results from the few population-based studies that do exist show that more women than men suffer from burnout and that age may be a factor of importance, but there is no consensus if higher age is positively or negatively related to burnout [21]. Moreover, underlying reasons for gender differences in burnout have not been extensively studied to our knowledge [8,18,19].

The aims of this cross-sectional study were to (1) assess the level of burnout in a representative sample from the actively working population in northern Sweden as measured by the SMBQ-instrument and (2) analyse relations to working conditions and gender, and interaction effects.

## Methods

In 1985 the northernmost counties in Sweden, Norrbotten and Västerbotten, became part of the World Health Organization MONICA (Multinational Monitoring of Trends and Determinants in Cardiovascular Disease) Project. The main goal of MONICA is to identify and register all stroke and myocardial infarction events in the participating areas [22]. Another part of the project is a recurrent screening of health status and cardiovascular disease risk factors in the general population. The screening includes a set of questionnaires and a clinical examination. The questionnaire was sent out together with an invitation letter to the clinical examination which was performed at the local healthcare centre. The questionnaire assessed health characteristics regarding cardiovascular disease, and relevant risk factors including work conditions and life-style. The Shirom Melamed Burnout Questionnaire (SMBQ) instrument was incorporated in the 2004 northern Sweden MONICA survey [23]. Ethical approval was granted by the Regional Ethical Review Board in Umeå (§464/03, dnr 03-375). Written informed consent was obtained from all participants.

In the present study we analyse information collected in the 2004 MONICA survey. Questionnaires were sent to a sample of 2500 persons aged 25-74 years (response rate 76.1%). The subjects were randomly chosen from the general population after stratification for sex and 10-year age intervals (25-34 years, 35-44 years, etc). Our study focuses on the actively working population and the following criteria were applied when selecting subjects for the analyses: age less than 65 years (430 excluded), occupationally active (453 excluded), and having answered all, or all but one, items in the SMBQ instrument (20 excluded). Participants who stated that they had a job and were not on sick leave were labelled as occupationally active. Those stating "sick leave" as their employment (144 respondents) were all excluded due to lack of information on the length of the sick leave period. The final study population consisted of 1000 participants (497 women and 503 men) from the Norrbotten and Västerbotten counties. The response rate for all respondents 25-64 years was 71.6%.

## Measurements

### Outcome

The Shirom Melamed Burnout Questionnaire (SMBQ) [23] consists of 22 items, each with a scale between 1-7 (1 being almost never and 7 almost always). "I feel tired", "my batteries are dead" and "I have trouble concentrating" are examples of items. An average mean score for each individual was calculated (score range 1-7). For subjects with a single missing answer, the missing value was replaced by the other respondents' median value on the particular question. Mean scores exceeding 4.0 indicate significant burnout symptoms [19]. In one study, high correlations were present between SMBQ and the Emotional Exhaustion subscale of the Maslach Burnout Inventory (MBI) and the Pines Burnout Measure [24]. The SMBQ instrument is however, in comparison to MBI, not as focused on the relationship between work and burnout. It focuses more on an overall mental and physical exhaustion in life [19]. In this paper, the individual SMBQ value is used to describe a person's level of burnout. Thus, the expressions "level of SMBQ" and "level of burnout" are used synonymously. A person with an SMBQ value exceeding 4 is classified as having "burnout".

### Background factors

Questionnaire information allowed for classification of standard socioeconomic index (SEI) for Sweden into five categories: blue-collar (unskilled), blue-collar (skilled), white-collar (low-medium), white-collar (high) and self-employed. Information on "work object group" according to the work object classification system developed by the MOA Research Group [25] was also collected. Work object group describes the type of work based on work title in three groups: working with people (teacher, care worker, etc.), working with things (mechanic, cook, etc.) or working with data (administrator, computer engineer, etc.). Information on "working hours" was available in three categories: fixed hours (for example, between 7 a.m. and 4 p.m. or constant night); shift work; and varying hours (sometimes day, sometimes evening, etc.). Number of work hours was divided into two groups, working between 1 and 35 hours a week (part-time) or working more than 35 hours a week (full-time).

Psychosocial work stress was assessed using the job demand and control dimensions of the demand-control model [26]. The Swedish version of the questionnaire contains 11 items divided into two groups: five questions measuring demand, and six measuring control. In the MONICA survey, two questions in the demand dimension differed from the original questionnaire: "Is your work physically heavy" and "Is your work psychologically trying". The original formulations were "Do you have to work hard?" and "Does your work demand great effort?". Participants with a demand score exceeding the 75^th ^percentile were classified as exposed to high demands, and participants with a control score below the 25^th ^percentile were classified as having low control.

"Physical work load" was dichotomized into sedentary or light work effort and work with modest or heavy activity. "Physical activity" during spare time was dichotomized into two groups, focusing on regular exercise. Those in the low activity group did not exercise or were mostly sedentary, whereas the exercise in the middle to high activity group ranged from walks a couple of times a week to more strenuous exercise.

Job insecurity was measured by two questions: "Are you at risk of becoming unemployed in the near future?" and "If you lost your job what would your chances be to get a new job within a month?" with alternative answers "very good", "rather good", "small" and "no chance".

"Social integration" was measured by the Availability of Social Integration (AVSI) instrument which is a part of the Interview Schedule of Social Interaction scale (ISSI) [27,28]. The abbreviated version of ISSI with six items was used. Questions included in AVSI are for example, "How many persons do you know who have the same interests as you?" and "How many friends can come home to you at anytime and feel at home?" with response alternatives; none, 1-2, 3-5, 6-10, 11-15 or more than 15. Results of the AVSI instrument, which assesses the respondent's quantitative social network, was dichotomized, based on earlier studies of risk groups, into high (3 or more friends) and low (fewer than 3 friends) social integration groups [29].

### Statistical analyses

The SMBQ was used as a continuous variable since the intention is to follow up this actively working population 5 years later and variations on the whole spectrum of the SMBQ scale will be of interest. It is also less likely to find many persons exceeding a clinically relevant cut-off point in an actively working population and therefore a high resolution scale is important.

The relation between SMBQ levels and various background factors were examined by one way ANOVA and multiple linear regression models. To select variables for the multiple regression model, an automated stepwise method was employed. This method has the ability to choose, step by step, the variables that are responsible for the largest portion of the explained variance. In each step the inclusion criterion was p-value < 0.05 and the exclusion criterion p-value > 0.10. Two variables, age and sex, were forced into the multiple linear regression. Interaction effects between sex and working conditions on the SMBQ level were tested in separate analyses of variance. Chi-square analyses were used for comparing proportions. Correlations were measured by calculating Pearson correlation coefficients. Two-tailed p-values were used for all hypothesis testing and the significance level was 0.05. SPSS version 14.0 was used for all analyses.

## Results

### Population and burnout

The prevalence of burnout in this actively working population was 12.9%. On average, women had a significantly higher level of SMBQ than men, Table 1. In analyses of separate age groups a significant difference between men and women was only found in the age group 35-44 years of age. Here 21.5% of the women had an SMBQ score exceeding 4.0. The SMBQ score was significantly related to young age in both sexes.

**Table 1:** Shirom Melamed Burnout Questionnaire (SMBQ) scores and percentage with SMBQ > 4.0 among men and women in different age groups

Age	Sex	N	Mean	p-value	Percentiles			SMBQ > 4.0	p-value
					**10**^**th**^	**50**^**th**^	**90**^**th**^	N (%)	
25-34	Men	103	2.89		1.82	2.73	4.07	13 (12.6%)	
	Women	116	3.10	(0.08)	2.04	3.05	4.31	19 (16.4%)	(0.43)
									
35-44	Men	138	2.77		1.73	2.62	4.14	16 (11.6%)	
	Women	130	3.09	0.01	1.78	2.98	4.55	28 (21.5%)	0.03
									
45-54	Men	145	2.78		1.71	2.59	4.04	15 (10.3%)	
	Women	149	2.95	(0.11)	1.68	3.00	4.14	22 (14.8%)	(0.25)
									
55-64	Men	117	2.57		1.45	2.55	3.74	6 (5.1%)	
	Women	102	2.75	(0.14)	1.77	2.45	4.15	10 (9.8%)	(0.19)
									
All ages	Men	503	2.75		1.68	2.59	3.98	50 (9.9%)	
	Women	497	2.98	<0.001	1.77	2.91	4.27	79 (15.9%)	0.005

SMBQ score range 1-7. Non significant p-values are in parentheses.

### Working conditions and burnout

In men, both high demands and low control contributed to higher levels of SMBQ, Table 2. Men with high job strain, a combination of high demand and low control, showed a mean SMBQ value of 3.42 (SD 0.95). Low self estimated risk for unemployment and good new job possibilities were associated with low SMBQ. Work object, socioeconomic class, working hours, number of work hours, and physical work load were not related to the SMBQ level in men.

**Table 2:** Shirom Melamed Burnout Questionnaire (SMBQ) scores related to working conditions

	MEN				WOMEN			
	N	Mean	SD	**p-value **^**a**^	N	Mean	SD	**p-value **^**a**^
**Work object**								
With data ^r^	128	2.73	0.86		131	2.81	0.85	
With things	236	2.74	0.91	(0.92)	68	3.28	1.12	0.002
With people	130	2.78	0.94	(0.75)	296	2.98	0.95	(0.17)
								
**Socioeconomic index (SEI)**								
Blue-collar (unskilled)	103	2.81	0.99	(0.23)	108	3.29	1.00	<0.001
Blue-collar (skilled)	121	2.76	0.91	(0.45)	86	2.96	0.92	(0.43)
White-collar (low-middle)	49	2.91	0.97	(0.10)	76	2.95	0.95	(0.33)
White-collar ^r ^(high)	168	2.67	0.86		199	2.83	0.91	
Self-employed	54	2.73	0.88	(0.68)	26	3.00	1.06	(0.40)
								
**Working hours**								
Fixed hours ^r^	329	2.76	0.92		316	2.89	0.96	
Shift work	59	2.86	0.85	(0.67)	43	2.98	0.95	(0.80)
Varying hours	110	2.64	0.88	(0.14)	136	3.20	1.02	0.001
								
**Number of work hours**								
Full-time	465	2.74	0.91		351	2.93	0.94	
Part-time (<35 hours)	30	2.82	0.87	(0.84)	140	3.11	1.03	(0.11)
								
**Demand at work**								
Low	359	2.65	0.86		340	2.89	0.93	
High	141	3.02	0.96	<0.001	155	3.19	1.01	0.001
								
**Control at work**								
High	396	2.71	0.90		358	2.91	0.92	
Low	105	2.92	0.92	0.03	137	3.18	1.04	0.004
								
**Physical work load**								
Modest - heavy	194	2.72	0.96		171	3.07	1.04	
Sedentary - light	307	2.72	0.87	(0.53)	325	2.94	0.92	(0.26)
								
**Risk for unemployment**								
Low	449	2.72	0.88		431	2.94	0.94	
High	53	3.03	1.06	0.02	63	3.30	1.05	0.004
								
**New job possibilities**								
Yes	291	2.63	0.86		276	2.93	0.99	
No	208	2.92	0.95	<0.001	219	3.05	0.92	(0.17)

^r ^Reference category. ^a ^Adjusted for age.

SMBQ score range 1-7. Non significant p-values are in parentheses.

In women, high demands and low control at work were associated with significantly higher SMBQ levels, Table 2. The highest SMBQ scores were found in women reporting high job strain situations (SMBQ mean 3.37, SD 1.19). Working "with things", being a blue collar worker, working varying hours and being at risk for unemployment were all associated with higher SMBQ values. In women who worked "with things" 76% also belonged to blue-collar unskilled workers. Physical work load, number of work hours, and new job possibilities did not significantly relate to SMBQ in women. Significant interaction effects were found between sex and work object (p < 0.05), and sex and working hours (p < 0.01). No interaction effects were found between sex and the other working conditions studied.

### Life situation and burnout

Marital status, children at home and smoking did not relate to SMBQ in either men or women, Table 3. A high level of social integration, high physical activity, and satisfaction with the economic situation were associated with low SMBQ in both sexes. More women than men reported a low social integration (women 23.7%, men 14.2%, p < 0.001), and were to a larger extent unsatisfied with their economic situation (women 30.6%, men 25.0%, p < 0.05). For women higher education was also related to a lower SMBQ level.

**Table 3:** Shirom Melamed Burnout Questionnaire (SMBQ) scores related to life situation

	MEN				WOMEN			
	N	Mean	SD	**p-value **^**a**^	N	Mean	SD	**p-value **^**a**^
**Marital status**								
Married or cohabiting	394	2.72	0.89		404	2.96	0.96	
Unmarried, divorced or widowed	101	2.91	0.96	(0.10)	87	3.08	0.97	(0.34)
								
**Living with children**								
Yes	270	2.80	0.93		286	3.03	0.96	
No	226	2.72	0.87	(0.62)	204	2.93	0.97	(0.99)
								
**Social support (integration)**								
High	424	2.68	0.88		377	2.86	0.93	
Low	70	3.20	0.97	<0.001	117	3.38	0.94	<0.001
								
**Physical activity**								
Medium - High	401	2.67	1.00		403	2.94	0.94	
Low	100	3.07	0.86	<0.001	94	3.17	1.04	0.02
								
**Smoker**								
No	440	2.74	0.89		384	2.96	0.95	
Yes	40	2.97	1.01	(0.07)	89	3.15	0.99	(0.07)
								
**Perceived economic situation**								
Satisfied	377	2.63	0.89		344	2.85	0.93	
Dissatisfied	126	3.10	0.88	<0.001	152	3.29	0.96	<0.001
								
**Education**								
University or likewise	139	2.72	0.80		195	2.86	0.90	
Others	362	2.76	0.94	(0.58)	299	3.06	0.99	0.02

^a ^Adjusted for age. SMBQ score range 1-7. Non significant p-values are in parentheses.

### Multiple linear regression analysis

Almost half (45%) of the gender difference could be explained in a stepwise multiple linear regression model containing lifestyle and work related factors, Table 4. The final model showed an R^2 ^value of 0.162. Included significant independent variables were age, three factors related to life situation; social integration, perceived economic situation, physical activity, and five work related factors; demand, control, physical work load, new job possibilities, and risk for unemployment.

**Table 4:** Multiple regression analyses of Shirom Melamed Burnout Questionnaire (SMBQ) scores and selected independent variables

	**Model **^**1**^		**Model **^**2**^		
	Beta	t-value	Beta	t-value	**R **^**2 †**^
**Age**	-0.123	-3.710***	-0.109	-3.213***	
**Sex**					
Male ^r^					
Female	0.108	3.253***	0.059	1.872	0.028
					
**Social support (integration)**					
High ^r^					
Low			0.187	5.874***	0.069
					
**Perceived economic situation**					
Satisfied ^r^					
Dissatisfied			0.138	4.230***	0.095
					
**Physical activity**					
Medium - high ^r^					
Low			0.131	4.180***	0.115
					
**Demand at work**					
Low ^r^					
High			0.166	5.140***	0.134
					
**New job possibilities**					
Yes ^r^					
No			0.078	2.289***	0.142
					
**Control at work**					
High ^r^					
Low			0.077	2.388*	0.154
					
**Physical work load**					
Modest - heavy ^r^					
Sedentary - light			0.070	2.127*	0.158
					
**Risk for unemployment**					
Low ^r^					
High			0.066	2.086*	0.162

F	15.369***		16.876***		

*P-value ≤ 0.05, **p-value ≤ 0.01, ***p-value ≤ 0.001. ^r ^Reference category. ^1 ^Variables entered: age and sex. ^2 ^Variables forced in: age and sex, variables entered stepwise: all independent variables from Table 2-3.

^† ^The combined R ^2 ^value from each step in the stepwise regression.

## Discussion

Burnout is often described as a condition that predominantly affects women. Our results do not, however, confirm that burnout is primarily related to female sex. In the multiple regression analysis the gender difference became non-significant when other factors were taken into account. In a previous Swedish study a high level of burnout was more common in women than men [8], but when adjusting for psychological distress and psychosocial work factors the gender effect disappeared, which is in accordance with our results.

In our study women working "with things" reported a higher level of burnout compared to women working "with people". These women working "with things" often belonged to low socioeconomic groups. Even though Maslach has incorporated different types of work in her burnout instrument, she originally concentrated the burnout concept on employees in people-related work. To our knowledge there are no previous data on the occurrence of burnout in different work object groups based on general population samples. The majority of previous studies on burnout have focused on specific subgroups who foremost were employed in the human service sector. Occupational differences in burnout prevalence may be explained to some extent by the level of job control [30].

The interaction effects found in this study between sex and work object, and sex and work hours, were explained by considerably higher SMBQ levels in women working "with things" and in women working "varying hours". In men no such variations were found for these variables. In separate analyses of men and women we found that low education level and low socioeconomic status were important factors for the level of burnout in women, but not in men. Blue collar work and a low level of education have correlated in some studies to burnout and especially for women [18,19]. Persons in insecure positions, for example temporary work, and those with lower socioeconomic status experience increased health risks [10]. Many blue-collar occupations also lack personal control over work.

Physical work load did not relate to SMBQ in women or men in univariate analyses, but in the multiple regression model physical work load revealed to have significant impact. High physical demands at work were negatively related to burnout. A plausible explanation is that jobs with high physical work load may have different effects and some may be beneficial for health.

Job insecurity, with risk for unemployment, and few new job potentials, also related to burnout in our study. Earlier results have shown associations between burnout and disagreeing about values at the work place [8]. This kind of work situation could be perceived as strenuous if the possibility to change the work place is slim. Social support from family and friends could be a stabilizer giving emotional and practical help to handle the situation.

Adverse life situation, such as low social integration, a perceived strained economic situation, and a low physical activity were associated to burnout. Social support has been shown to correlate with burnout and work related outcomes [31]. An unexpected result in our study was that low social integration was more common among women than men. Previous research on social support has reported that women had more friends and consequently received more emotional support from their social networks [32].

In our actively working population the SMBQ level was negatively associated with increasing age. There are conflicting results from previous studies concerning the effect of age on burnout symptoms [8,18,19,33]. Some of the varied results may be due to the fact that various samples are differently selected, and that only few studies have included samples from the general working population. The response rate in population based surveys may also influence the results. In many survey studies young people have been less willing to participate than the older. The "healthy worker effect" could be a confounder in our study since elderly people who remain in the working force may be healthier than their younger counterparts and those who do not work due to sick leave. An escalating burnout risk with increasing age has been found in studies using the MBI-GS instrument [8,18]. Some of the varied results may be due to different properties of the burnout instruments used. The SMBQ instrument is a more overall burnout instrument which can be used also for persons outside the labour market, while the MBI-GS is only applicable on strict work-related burnout.

The highest SMBQ scores in our study were found in younger persons. They are supposed to find a place in life, maybe raise a family and build a professional career. The balancing between home and work life can be hard, especially if the individuals are exposed to many risk factors or strains simultaneously. In Sweden, the situation for young persons has deteriorated during the last decade, probably mostly due to difficulties in entering the labour market and more insecure employments [34].

### Study limitations and strengths

The multiple regression model in our study explained about 16% of the SMBQ variance which may be considered as low. In earlier studies burnout has been shown to have strong connections to personality factors and to other physical or psychological illnesses [19,35]. These factors often co-vary strongly with burnout and therefore they were not included in our analyses. A previous study on burnout, controlling for negative affectivity, implied significant associations between work related factors and burnout [13]. It is important to highlight that our definition of burnout is wide and might encompass not only work related burnout.

The high response rate, exceeding 75 percent, in the 2004 MONICA survey is a strength in this study. In addition, the age and sex group distributions were approximately equal which is favourable when gender aspects are in focus. The population consisted of a randomly chosen sample from the general population and was homogeneous in the respect that everybody was actively working and different occupations were represented.

This study was based on cross sectional data and this implies difficulties in drawing conclusions on causalities. A high social support or a low physical activity may be either a cause or a consequence of burnout. Longitudinal data are required to provide information on risk factors for the development of burnout. We plan to investigate this cohort further to study how burnout symptoms develop over time.

## Conclusions

In this study of actively working people from the general population in northern Sweden the prevalence of burnout was 13%, with a significantly higher rate for women than for men (women 16%, men 10%). A model containing both work related factors and life situational factors explained approximately half of the sex difference. In women, the work related factors which showed significant associations to burnout were to a large extent related to the socioeconomic situation. This association needs to be considered in further studies on burnout.

A high prevalence of burnout in the working population will probably have large negative impact on the productivity and welfare in society at large. In the OECD countries disability pensions due to poor mental health and burnout have increased [36-38]. This situation requires preventive strategies which, according to our results, should include measures to improve working conditions and related socioeconomic factors especially for women. Regular assessments by the occupational health services and early identification of increased risk for future disability will be of importance [37].

## Competing interests

The authors declare that they have no competing interests.

## Authors' contributions

The study was conceived and designed by all authors. SN performed all analyses and drafted the paper. All authors provided key intellectual input and edited the paper. LSB obtained funding. All authors read and approved the final manuscript.

## Pre-publication history

The pre-publication history for this paper can be accessed here:

http://www.biomedcentral.com/1471-2458/10/326/prepub
